# Molecular Epidemiology of Isoniazid-resistant *M tuberculosis* in Port-au-Prince, Haiti

**DOI:** 10.1093/ofid/ofae421

**Published:** 2024-07-18

**Authors:** Kathleen F Walsh, Myung Hee Lee, Chrispin Chaguza, Widman Pamphile, Gertrude Royal, Vincent Escuyer, Jean W Pape, Daniel Fitzgerald, Ted Cohen, Oksana Ocheretina

**Affiliations:** General Internal Medicine, Weill Cornell Medicine, New York, New York, USA; Center for Global Health, Weill Cornell Medicine, New York, New York, USA; Center for Global Health, Weill Cornell Medicine, New York, New York, USA; Department of Epidemiology of Microbial Diseases, Yale School of Public Health, Yale University, New Haven, Connecticut, USA; Groupe Haitian d'Etude du Sarcoma da Kaposi at des Infections Opportunistas (GHESKIO), Port-au-Prince, Haiti; Groupe Haitian d'Etude du Sarcoma da Kaposi at des Infections Opportunistas (GHESKIO), Port-au-Prince, Haiti; Wadsworth Center, New York State Department of Health, Albany, New York, USA; Center for Global Health, Weill Cornell Medicine, New York, New York, USA; Groupe Haitian d'Etude du Sarcoma da Kaposi at des Infections Opportunistas (GHESKIO), Port-au-Prince, Haiti; Center for Global Health, Weill Cornell Medicine, New York, New York, USA; Department of Epidemiology of Microbial Diseases, Yale School of Public Health, Yale University, New Haven, Connecticut, USA; Center for Global Health, Weill Cornell Medicine, New York, New York, USA

**Keywords:** adolescent, epidemiology, isoniazid, resistance, tuberculosis

## Abstract

**Background:**

Isoniazid-resistant, rifampin-susceptible tuberculosis (Hr-TB) is associated with poor treatment outcomes and higher rates of acquisition of further drug resistance during treatment. Due to a lack of widespread diagnostics, Hr-TB is frequently undetected and its epidemiology is incompletely understood.

**Methods:**

We studied the molecular epidemiology of Hr-TB among all patients diagnosed with culture-positive pulmonary tuberculosis between January 1 and June 30, 2017, at an urban referral tuberculosis clinic in Port-au-Prince, Haiti. Demographic and clinical data were extracted from the electronic medical record. Archived diagnostic *Mycobacterium tuberculosis* isolates were tested for genotypic and phenotypic isoniazid resistance using the Genotype MTBDR*plus* assay (Hain, Nehren, Germany) and culture-based testing, respectively. All isoniazid-resistant isolates and a randomly selected subset of isoniazid-susceptible isolates underwent whole-genome sequencing to confirm the presence of mutations associated with isoniazid resistance, to validate use of Genotype MTBDR*plus* in this population, and to identify potential transmission links between isoniazid-resistant isolates.

**Results and Conclusions:**

Among 845 patients with culture-positive pulmonary tuberculosis in Haiti, 65 (7.7%) had Hr-TB based on the Genotype MTBDR*plus* molecular assay. Age < 20 years was significantly associated with Hr-TB (odds ratio, 2.39; 95% confidence interval, 1.14, 4.70; *P* = .015). Thirteen (20%) isoniazid-resistant isolates were found in 5 putative transmission clusters based on a single nucleotide polymorphism distance of ≤ 5. No patients in these transmission clusters were members of the same household. Adolescents are at higher risk for Hr-TB. Strains of isoniazid-resistant *M tuberculosis* are actively circulating in Haiti and transmission is likely occurring in community settings.

## Teaser

The overall proportion of tuberculosis in Haiti that is isoniazid resistant is 7.7%; a higher proportion of resistance in younger cases suggests a rise in the fraction of incident tuberculosis that is resistant. Genomic analyses suggest transmission of isoniazid-resistant *Mycobacterium tuberculosis* in community settings.

Isoniazid (INH) resistance is the most common type of drug resistance in *Mycobacterium tuberculosis* (Mtb) among standard first-line drugs [1]. Diagnostics to detect INH resistance are expensive and thus often unavailable in low-resource settings. When INH resistance is undetected and individuals with isoniazid-resistant tuberculosis (Hr-TB) are treated with drug-susceptible regimens, they experience higher rates of acquired drug resistance, treatment failure, and death compared to those with drug-susceptible tuberculosis [2, 3]. INH resistance is often a critical early step along the pathway to acquisition of the multidrug-resistant phenotype [4, 5].

To determine the prevalence of and risk factors for Hr-TB, we retrospectively tested archived Mtb isolates from all patients diagnosed with culture-positive pulmonary tuberculosis for INH resistance during a 6-month period in 2017 at an urban tuberculosis clinic in Port-au-Prince, Haiti.

## METHODS

### Study Setting and Population

This study was conducted at GHESKIO in Port-au-Prince, Haiti. GHESKIO is the largest tuberculosis treatment center in Haiti. Haiti comprises 10 geographic departments; the West department, which includes Port-au-Prince, has the highest population density. Based on a spatial analysis of Haiti's National TB Program surveillance data, GHESKIO diagnoses approximately 10% of all TB in Haiti and 24% of all TB diagnosed in the Port-au-Prince metro region [6].

All patients who present to GHESKIO with symptoms suggestive of tuberculosis have spot and early morning sputum examined for Mtb by the Xpert MTB/RIF (Cepheid, Sunnyvale, CA) molecular assay. If a sputum sample is positive for Mtb on Xpert, the sample then undergoes liquid culture in the BACTEC system in an on-site BSL3 laboratory. GHESKIO diagnoses approximately 1500 culture-positive tuberculosis cases annually. It serves as the main referral TB laboratory for Haiti.

All patients at GHESKIO between January 1, 2017 and June 30, 2017, diagnosed with pulmonary tuberculosis based on clinical symptoms, chest radiograph consistent with pulmonary tuberculosis, and positive for Mtb by the Xpert MTB/RIF molecular assay without evidence of rifampin resistance at time of diagnosis and with an archived diagnostic Mtb culture isolate were included in this study.

Persons with strains of Mtb resistant to rifampin were excluded because the epidemiology of rifampin-resistant tuberculosis is better understood in Haiti and outside the focus of this investigation.

### Study Design

This was a retrospective, cross-sectional study designed in 2019 of the prevalence of isoniazid resistance among patients diagnosed with culture-positive rifampin-susceptible pulmonary tuberculosis at GHESKIO in Port-au-Prince, Haiti in 2017. GHESKIO routinely cultures all diagnostic sputum samples that are positive for Mtb based on the Xpert MTB/RIF molecular assay and routinely archives isolates from all positive cultures at −80 °C. Archived diagnostic culture isolates from patients diagnosed with pulmonary tuberculosis between January 1 and June 30, 2017, were tested for isoniazid resistance using the Genotype MTBDR*plus* molecular assay (Hain Lifescience, Nehren, Germany). All isolates determined to be isoniazid resistant by this molecular assay and an equal number of randomly selected isoniazid-susceptible isolates to serve as controls underwent phenotypic and genotypic drug susceptibility testing.

To confirm that the Genotype MTBDR*plus* assay was detecting mutations associated with phenotypic INH resistance in this population, DNA from all INH-resistant isolates and an equal number of randomly selected INH-susceptible isolates that underwent culture-based drug susceptibility testing all underwent whole-genome sequencing. DNA was extracted from all INH-resistant isolates and the same equal number of INH-susceptible isolates that underwent drug susceptibility testing and shipped for whole-genome sequencing to the Wadsworth Center of the New York State Department of Health. Clinical and demographic information were extracted from the electronic medical record. For each patient with TB, GHESKIO staff documents household contacts; this was extracted from the medical record to determine if patients were in the same household.

### Laboratory Procedures

Banking of Mtb isolates: Sputum samples were adjusted to 5 mL with sterile water and decontaminated with 5 mL of NALC-NaOH (3% sodium hydroxide, 0.5 to 0.6% N-acetyl-L-cysteine, 1.47% sodium citrate). Automated liquid culture was performed using the BACTEC MGIT 960 instrument (Becton and Dickenson, Franklin Lakes, NJ, USA) in accordance with the manufacturer's instructions. The presence of Mtb in positive BACTEC cultures was confirmed with the SD-Bioline rapid MPT64 test (Standard Diagnostics, Yongin, Korea). All Mtb isolates are routinely preserved by freezing in 7H9 Middlebrook media supplemented with 15% glycerol at –80 ° C in the GHESKIO laboratory.

Molecular drug susceptibility testing: Fifty microliters of preserved Mtb isolate were transferred into a microtube with 300 µL of nuclease-free water. Bacteria were heat killed by incubation at 80 °C for 1 hour. DNA was released with 15 minutes of sonication in an ultrasound bath and the resulting crude extract was separated from debris by centrifugation and tested directly with the Genotype MTBDR*plus* line probe assay in accordance with the manufacturer's instructions [7].

Phenotypic drug susceptibility testing: Mtb strains found resistant to INH by the Genotype MTBDR*plus* assay and an equal number of randomly selected INH-susceptible strains, also based on the Genotype MTBDR*plus* assay, were regrown in MGIT 960 tubes and tested for resistance to 0.1 µg/mL of INH by MGIT 960 SIRE assay (Becton Dickenson) according to the manufacturer's instructions.

Isolation of genomic DNA: Frozen Mtb isolates were thawed, and 0.1 mL was inoculated into a BACTEC MGIT tube and incubated at 37 °C for 3–7 days to obtain sufficient material for genomic DNA preparation. Following incubation, 1.5 mL bacterial suspension from the bottom of the MGIT tube was transferred into a microtube and heat-killed for 1 hour at 80 °C. Cell material was collected by centrifugation for 15 minutes at 15,000*×g*, then resuspended in 200 µL of the InstaGene matrix (BioRad, Hercules, USA) and incubated for 30 minutes at 56 °C. Contents of the tube were transferred into a screw-cap XXTuff reinforced microtube with 0.1-mm zirconia/silica beads (Biospec Products, Bartlesville, USA), vortexed for 10 seconds and then incubated for 20 minutes at 100 °C. Mtb cells were disrupted by bead-beating for 30 seconds at maximum speed in a D1030 BeadBug microtube homogenizer (Benchmark Scientific, Sayreville, USA). After 15 minutes’ centrifugation at 15,000*×g*, 100 µL of supernatant containing purified genomic DNA were collected into a clean microtube.

Whole-genome sequencing (WGS): Genomic Mtb DNA was taken from INH-resistant and INH-susceptible isolates. DNA concentration of samples was between 0.4 and 21 ng/µL. Sequencing libraries were prepared using the Illumina DNA Prep, (M) kit and IDT for Illumina DNA/RNA UD Indexes, Tagmentation. Final libraries were quantified using the Quant-it HS DNA Kit (Invitrogen, Waltham, USA), pooled and sequenced on an Illumina NextSeq 500/550 using the NextSeq Mid Output kit, v2 (300 cycle), 2 × 149-bp paired end reads. FASTQ files were generated using the bcl2fastq v2.20.0.422 program (https://support.illumina.com/sequencing/sequencing_software/bcl2fastq-conversion-software/downloads.html). All samples generated at least 50× genome coverage sufficient for data analysis.

### Data Analysis

Clinical and demographic data: The association between individual-level covariates and INH resistance were first examined using a univariable logistic model. Covariates included age, sex, HIV status, prior INH use, history of TB, and neighborhood of primary residence. Variables statistically significant in univariable analysis and those judged to be clinically significant were included in a multivariable logistic model. Potential interactions between covariates in the multivariable model were also examined.

Whole-genome alignment and lineage assignment: Whole-genome alignments of the sequenced Mtb strains were generated using snippy (V4.6.0) pipeline for fast variant calling and alignment of bacterial sequencing data (https://github.com/tseemann/snippy). The complete reference whole-genome sequence of Mtb H37Rv strain [8] was used as the reference for the variant calling and whole-genome alignment (GenBank accession number: NC_000962.3). To automatically mask problematic genomic sites in the whole-genome alignment before downstream analysis, a custom browser extensible data file containing H37Rv genomic coordinates of the sites to mask was provided to the snippy-core command using the “–mask” option (https://github.com/ChrispinChaguza/MTB_HAITI). Lineages were assigned based on the Coll [9], Freschi [10], Lipworth [11], Shitikov [12], and Stucki [13] genotyping schemes using fast-lineage-caller (https://github.com/farhat-lab/fast-lineage-caller). Associations between drug resistance profiles and drug resistance-conferring mutations were visualized using chord diagrams plotted by the “chordDiagram” function in circlize (version 0.4.15) R package [14].

Phylogenetic analysis: Single nucleotide polymorphism (SNP) alignments and SNP distance matrices were produced using snp-sites (version 2.5.1) [15] and snp-dists [V0.7.0] (https://github.com/tseemann/snp-dists). The maximum likelihood tree was calculated using the SNP alignment with IQTREE (V2.0.3) using the best-fit model selection option [15]. We ran IQTREE using the “-fconst” option with the following values “702044,1324366,1318957,701346” generated by snp-sites (V2.5.1) [15]. The *Mycobacterium bovis* BCG Moreau RDJ strain (GenBank accession number: AM412059) was used as an outgroup to root the phylogenetic tree of the Mtb strains. Processing and manipulation of the generated phylogenetic tree, including outgroup-based rooting, ladderizing the tree, dropping the outgroup from the generated rooted tree, and visualizing done using ape (V 5.6.2) [16] in R (V4.0.3) [https://cran.r-project.org/] and ggtree (V3.10.0) [17]. Antimicrobial resistance profiles were predicted using Mykrobe (V0.11.0) [18].

The frequency of Mtb lineages based on the Coll [9] scheme among INH-resistant and INH-susceptible strains was assessed using the chi-squared test. The distribution of the Mtb sublineages among the INH-resistant and INH-susceptible isolates were assessed using the Simpson index of diversity. The statistical significance and 95% confidence intervals (CI) for the Simpson index of diversity were estimated using the resampling approach.

Putative transmission analysis: Genetic relatedness between pairs of strains was determined by the number of SNPs distinguishing the strains across the whole genome after masking problematic genomic sites, including those containing repetitive sequences that may not evolve neutrally and may bias phylogenomic analyses. A strict distance of ≤5 SNPs as well as a less conservative SNP distance of ≤12 was used to define genetic clusters containing related strains, which may be part of a relatively recent transmission chain [19–23]. Pairs of strains with SNP distances less than the specified maximum SNP distance threshold were joined by an edge to construct a network showing the connectedness of the sequenced strains to infer potential transmission clusters. The network was constructed and visualized using igraph (V1.2.6) package [24] in R. Once transmission clusters were determined based on sequencing data, household contact data extracted from the medial record was examined to determine if participants represented in clusters were from the same household.

### Ethical Oversight and Patient Consent Statement

This study was approved by the Weill Cornell Medicine and GHESKIO institutional review boards. It was a retrospective study of laboratory data and written patient consent was not required.

## RESULTS

**Figure 1.**
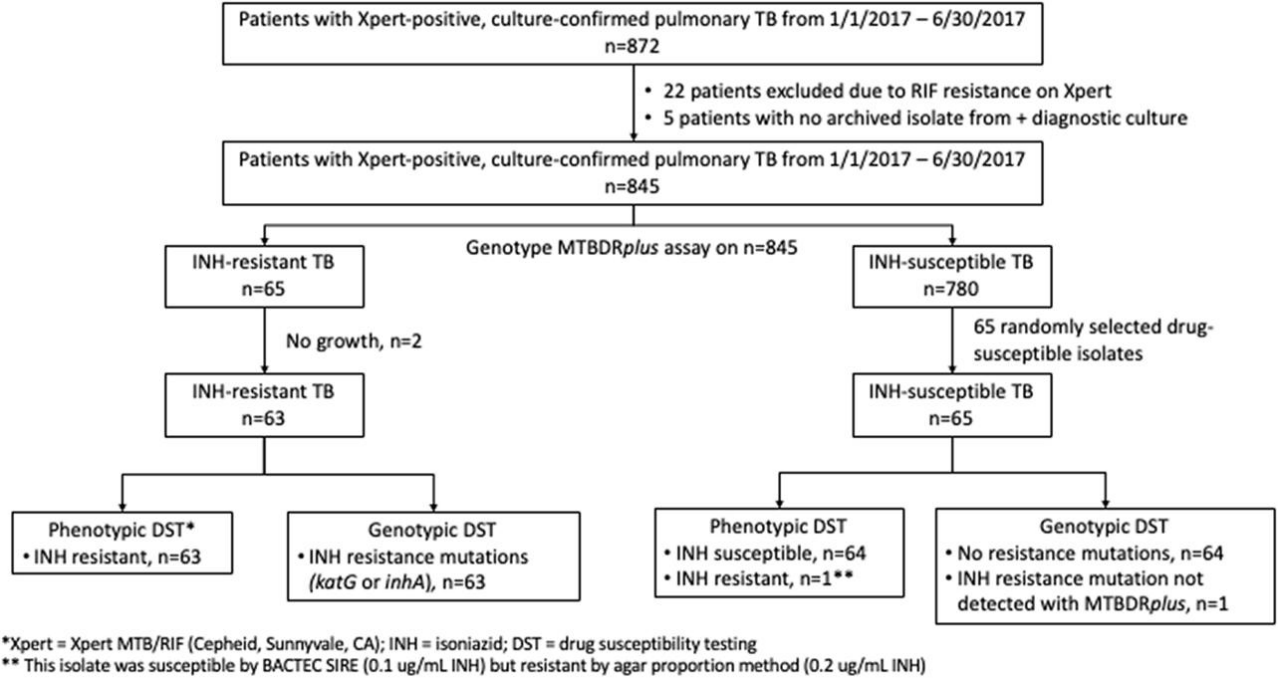
Study population.

Between January 1, 2017, and June 30, 2017, 872 patients were diagnosed with Xpert-positive, culture-positive pulmonary tuberculosis at GHESKIO (Figure 1). Twenty-seven patients were excluded from the final study cohort because of rifampin resistance on Xpert MTB/RIF (n = 22) or no archived diagnostic culture isolate (n = 5).

The final study population included 845 patients diagnosed with Xpert-positive, culture-positive pulmonary tuberculosis without evidence of rifampin resistance. One hundred and eleven (13%) patients had HIV (Table 1). Ninety-three patients (11%) were adolescents <20 years old, and all of these adolescents were HIV negative. Of the 845 patients, 746 (89%) were presenting with a new case of tuberculosis and 99 (11%) had been previously treated for tuberculosis (it was not determined if these subsequent episodes were recurrence or relapse).

**Table 1.** Demographic and Clinical Characteristics of 845 Patients With Pulmonary Tuberculosis in Port-au-Prince, Haiti

Characteristic	Value
Sex, n (%)	…
Male	477 (56)
Female	368 (44)
Age, median [IQR]	29 [23, 39]
Age <20 y^a^, n (%)	93 (11)^a^
Age ≥20 y, n (%)	752 (89)
Education, n (%)	…
Primary school or less	342 (40)
Secondary school or higher	466 (55)
Missing data	37 (5)
Marital status	…
Single	507 (60)
Married or living together	240 (29)
Divorced or separated	45 (5)
Widower	17 (2)
Missing data	36 (4)
Positive HIV status, n (%)	111 (13)
Type of TB, n (%)	…
New case	746 (88)
Previously treated	99 (12)
Xpert^b^ level, n (%)	…
Very low	63 (7)
Low	147 (17)
Medium	375 (44)
High	260 (32)

Abbreviations: IQR, interquartile range; TB, tuberculosis.

^a^None of the adolescents (age < 20) were HIV seropositive.

^b^Xpert MTB/RIF (Cepheid, Sunnyvale, CA).

### Prevalence

All isolates were screened for the presence of mutations in the 2 main molecular determinants of INH resistance by the Genotype MTBDR*plus* line probe assay: S315 codon of the *katG* gene and the *mabA-inhA* promoter region. Based on this assay, 65 of 845 (7.7%) patients had Hr-TB. Of 746 patients with their first episode of tuberculosis, 55 (7.4%) had Hr-TB; 10 of 99 patients (10.1%) presenting with a previously treated case of tuberculosis had Hr-TB.

### Clinical and Demographic Covariates

**Figure 2.**
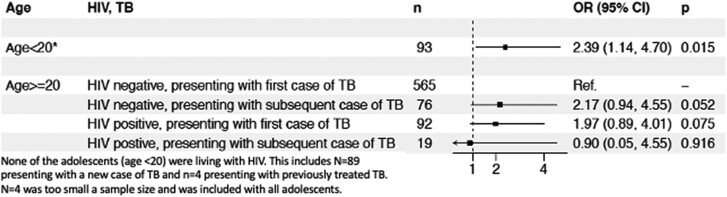
Multivariate model of predictors of isoniazid-resistant TB among 845 pulmonary tuberculosis patients in Port-au-Prince, Haiti, with forest plot depicting odds ratio of isoniazid-resistant TB among groups.

In univariable models, age <20 years was the only significant covariate (odds ratio, 1.95; 95% CI, 0.96–3.69; *P* = .049) (Supplementary Table 1). Age <20 years, HIV status, and type of TB (new vs previously treated) were included in the multivariable model. In this model, patients aged <20 years had 2.39 greater odds (95% CI, 1.14–4.70; *P* = .015) of having Hr-TB compared to the reference group (new TB cases, HIV negative, aged ≥ 20 years) (Figure 2). Of note, in a subanalysis of only those with HIV, prior INH monotherapy within the context of preventive therapy among those with HIV did not impact the odds of having Hr-TB.

### Phenotypic Drug Susceptibility Testing by Bactec ACTEC Liquid Culture

Of the 65 INH-resistant isolates identified by the Genotype MTBDR*plus* assay, 2 isolates did not grow and were not included in further analysis (Figure 1). Sixty-three INH-resistant isolates were tested for phenotypic drug susceptibility using the MGIT 960 SIRE assay in BACTEC liquid culture system. All 63 isolates (100%) were phenotypically resistant at an INH concentration of 0.1 µg/mL in liquid culture. Of the 65 randomly selected INH-susceptible isolates based on the Genotype MTBDR*plus* assay, all 65 were susceptible to INH at 0.1 µg/mL in the MGIT 960 SIRE assay.

Regarding the other first-line drugs, all 128 isolates were susceptible to rifampin 1.0 µg/mL, ethambutol 5.0 µg/mL, and pyrazinamide 100 µg/mL. Two isolates were resistant to streptomycin 1.0 µg/mL (1 INH-resistant and 1 INH-susceptible) and 1 INH-resistant isolate had intermediate susceptibility.

### Genotypic Drug Susceptibility Testing by WGS

WGS was performed on DNA extracted from 63 INH-resistant Mtb culture isolates and 65 INH-susceptible Mtb culture isolates, as determined by the Genotype MTBDR*plus* assay. Of the 63 INH-resistant strains, 17 (27%) had a C-15T *mabA-inhA* promoter mutation; 1 had an additional high-confidence S94A mutation in the *inhA* coding region. The remaining 46 strains had 4 different polymorphisms in the S315 *katG* codon: 43 (68%) with *katG* S315T (AGC -> ACC); 1 (2%) with *katG* S315T (AGC -> ACA); 1 (2%) with *katG* S315N (AGC -> AAC); 1 (2%) with a *katG* deletion.

Of the 65 successfully sequenced isolates in the INH-susceptible group, 1 isolate in sublineage 4.1.2.1 [9] was found to harbor a synonymous L203L mutation in the *mabA* gene. This mutation is known to be associated with INH resistance [25] but the Genotype MTBDR*plus* assay is not designed to detect it. This isolate was susceptible to INH at the 0.1 µg/mL concentration when tested in liquid culture. Following the sequencing results, this isolate was additionally tested on 7H10 Middlebrook solid media with Agar Proportion method as recommended [26]. The isolate was found to be resistant to the low concentration of INH (0.2 µg/mL) but sensitive to the high concentration (1 µg/mL) of INH on solid media.

### Phylogenetic Tree and Putative Transmission Clusters

**Figure 3.**
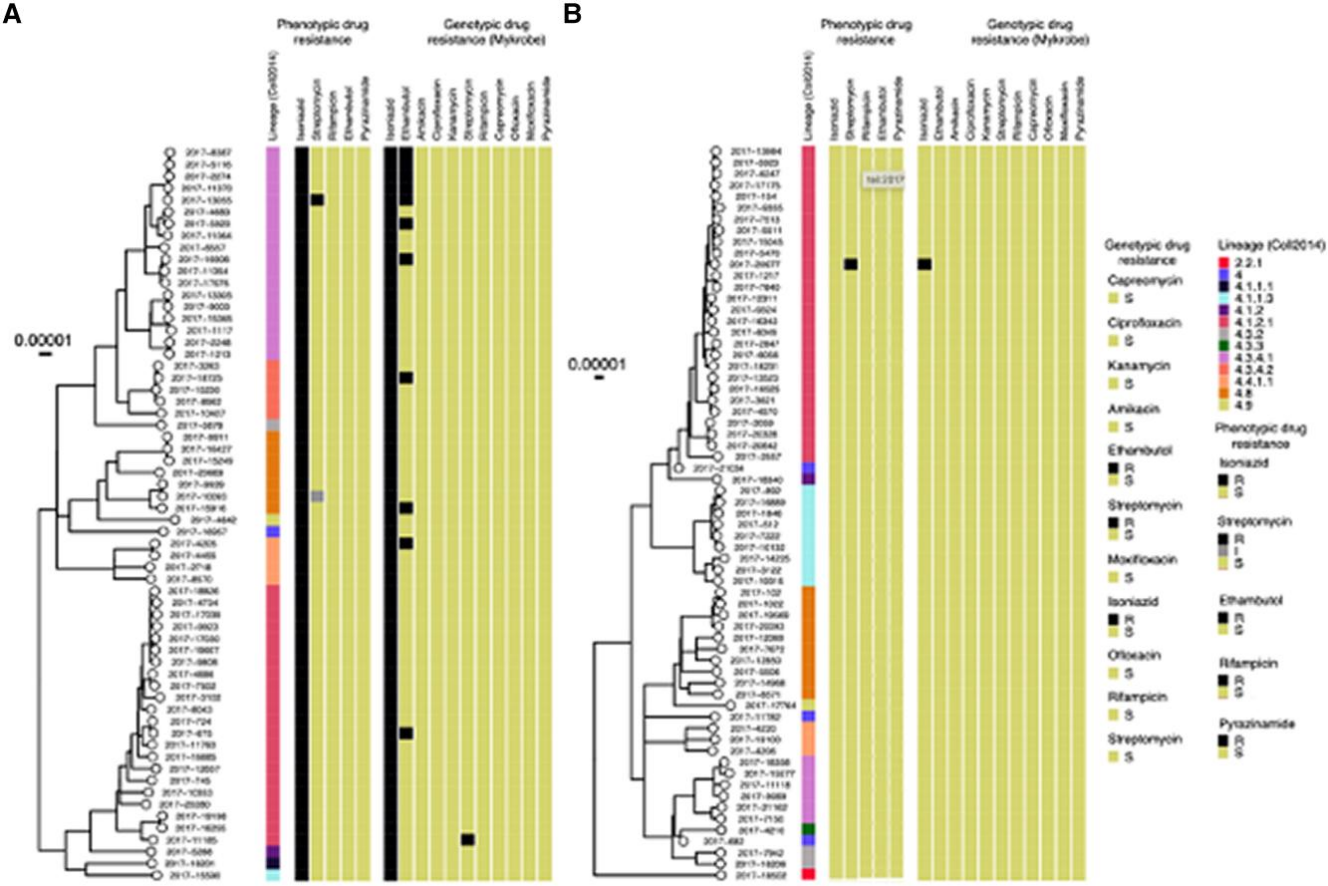
Phylogenetic tree with lineage, phenotypic and genotypic drug resistance among isoniazid-resistant strains (*A*) and isoniazid-susceptible strains (*B*) as determined by Genotype MTBDR*plus* assay.

Of the 128 isolates sequenced, n = 127 were from the Euro-American lineage (Coll lineage 4) [9]. One INH-susceptible isolate was from the East Asian lineage (Coll lineage 2) (Figure 3, Supplementary Figure 1). The majority of both INH-susceptible and INH-resistant isolates were from Coll sublineage 4.1.2.1 (Supplementary Table 2, Supplementary Figure 2). A high proportion of INH-resistant isolates were from sublineage 4.3.4.1 (INH-resistant:18/63, INH-susceptible: 6/65, *P* = 0.011).

**Figure 4.**
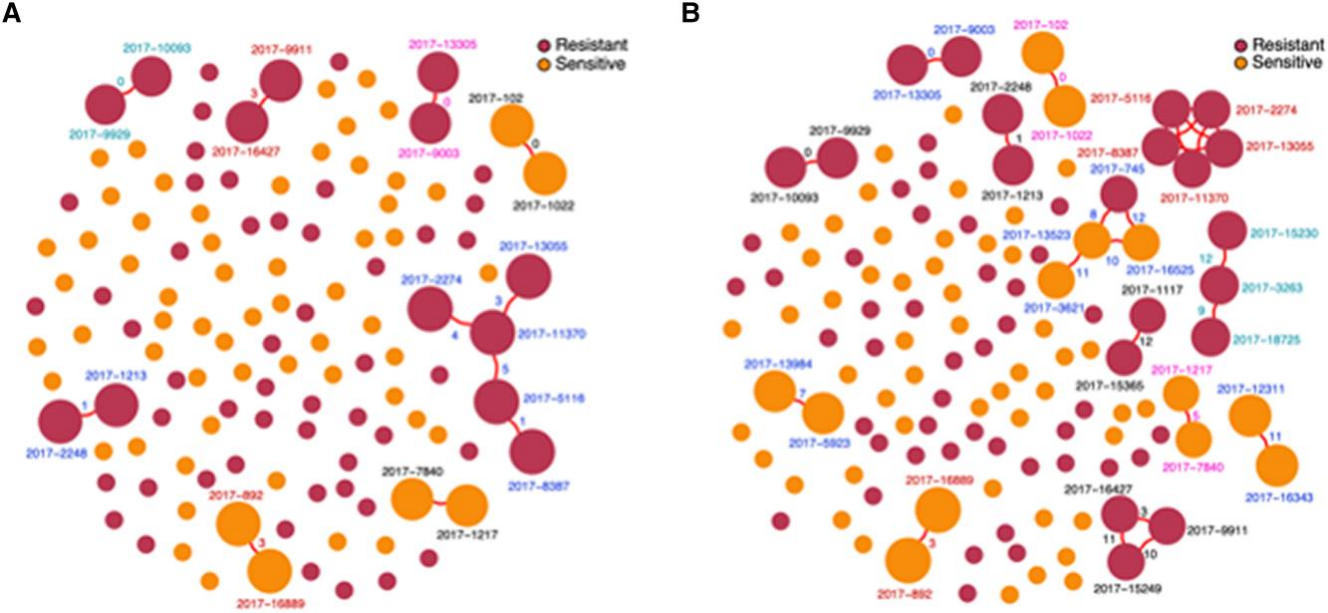
Putative transmission clusters of *M tuberculosis* strains in Port-au-Prince, Haiti, stratified by isoniazid resistance as determined by Genotype MTBDRplus. (*A*) Clusters determined by a single nucleotide polymorphism (SNP) threshold of < 5. (*B*) Clusters determined by a SNP threshold of < 12.

The WGSs from the generated alignment with masked repetitive and other hypervariable sites were compared to determine the genetic distance, based on the number of SNPs, between pairs of isolates. Overall, the number of SNPs found among INH-resistant strains was slightly higher than that among the INH-susceptible strains of *M tuberculosis* (*P* = .00003, Kruskal-Wallis test) (Figure 4).

Using an SNP distance of ≤5, there were 5 transmission clusters of INH-resistant isolates (13/63, 20%) (Figure 4). Of the 13 clustered INH-resistant isolates, 12 (92%) were from patients with a new diagnosis of tuberculosis; 1 isolate was from a patient who had been previously treated for tuberculosis. Two of the 13 clustered INH-resistant isolates belonged to patients aged <20 years (15%) and 2 isolates belonged to adults with HIV (15%).

Using an SNP distance of ≤ 12, there were 8 transmission clusters of INH-resistant isolates (19/63, 30%) and 5 clusters of INH-susceptible isolates (10/65, 15%), with 1 cluster including both susceptible and resistant isolates. We caution that the clustering rate of INH-susceptible isolates should not be compared with that of the INH-resistant isolates because of the different sampling coverages imposed by our study design.

## DISCUSSION

We found that 7.7% of all pulmonary tuberculosis cases in Port-au-Prince, Haiti, are Hr-TB, 7.4% among those with a new case and 10.1% among those previously treated. This is comparable to the global average reported by the World Health Organization [1]. Adolescents (age < 20 years) had more than 2-fold increased odds of having Hr-TB compared to adults aged 20 years or older. This strong association with younger age suggests a higher proportion of Hr-TB in recently transmitted Mtb strains in Haiti. Twenty-two percent of INH-resistant Mtb isolates from patients with a new diagnosis of tuberculosis were in 5 distinct molecular clusters consistent with ongoing transmission of Hr-TB. None of the clusters was within households, suggesting transmission in other community settings.

Adolescents are uniquely at risk for tuberculosis because of both increased epidemiologic and immunologic susceptibility to Mtb infection [27–29]. Studies examining the natural course of tuberculosis from the prechemotherapy age also suggest adolescence may be a high risk time for rapid progression from Mtb infection to disease onset [27, 30–32]. Once symptomatic, they are also at risk for transmitting Mtb because of frequent delays in diagnosis, large social networks, and high number of contacts [31, 33–35]. Younger age has been associated with Hr-TB in other settings as well [36, 37]. Given adolescents’ younger age, Mtb strains that cause disease in adolescents most likely represent currently circulating strains, compared to adults with tuberculosis who may have been infected with Mtb decades earlier. The higher proportion of Hr-TB in adolescents in Haiti suggests that the transmission of INHr strains may be increasing.

People with HIV presenting with their first episode of tuberculosis may demonstrate a similar phenomenon because they are also at elevated risk of rapid progression from Mtb infection to symptomatic disease [38]. In essence, both adolescents and people with HIV may act as “canaries in the coal mine” and may be harbingers of emerging INH resistance. Of note, there was no association between the use of INH preventive therapy and Hr-TB in our patients with HIV.

Identifying sentinel groups that may predict future prevalence of drug-resistant TB would be of great benefit for resource-limited settings, which many TB-endemic countries are. It would enable TB programs to direct surveillance efforts at a small group of patients who would provide critical data to prevent widespread transmission of certain Mtb strains. In situations of civil unrest such as that facing Port-au-Prince, this would enable limited resources to be put to their best use.

Twenty-two percent of INH-resistant Mtb isolates from patients with a new diagnosis of tuberculosis were in 5 distinct molecular clusters. These clusters were not among household contacts suggesting community transmission outside the home. This is consistent with studies demonstrating that a significant proportion of Mtb transmission occurs in community settings outside the household [39, 40]. A large proportion of the INH-resistant strains in our study were from the same sublineage, Coll 4.3.4.1. Although our study design does not allow for strict comparison between these groups, it may suggest that certain Mtb sublineages are enriched for drug resistance.

This study used data collected through routine medical records. Thus, the analysis of transmission clusters was based solely on sequencing data, which limits a full understanding of Hr-TB transmission in Port-au-Prince. Data on household contacts extracted from the medical record allowed for determination that no patients represented in molecular clusters were from the same household although direct epidemiologic links may have been missed. We included strains collected over a short time frame, which also limits the ability to fully describe the extent of Hr-TB transmission in Port-au-Prince. Treatment outcomes were outside the scope of this study and may have provided additional insight into the importance of Hr-TB detection in this population. Future research is needed to prospectively investigate epidemiologic links between Hr-TB cases and identify any changes in Hr-TB epidemiology that may have occurred since 2017, the year when the patients in this study were diagnosed.

## CONCLUSION

This study demonstrates that adolescents may be sentinel groups for Hr-TB, raising concerns about an emerging epidemic of this form of drug resistance. Adolescents should be prioritized for screening for INH resistance.

## Supplementary Material

ofae421_Supplementary_Data

## Notes

***Acknowledgments*.** We gratefully acknowledge the Wadsworth Core Facility, especially Pascal Lapierre, Fondation Merieux, and the dedicated laboratory and clinical staff at GHESKIO who work tirelessly to provide enhanced tuberculosis testing and care for Haiti.

***Financial support***. K. F. W. is supported by NIAID K23 AI175686.

***Author contributions:*** K. F. W.: study design, data collection & analysis, wrote the manuscript; M. H. L.: data analysis, manuscript writing and critical review; C. G.: data analysis specifically all genomic analyses, manuscript writing and critical review; W. P. and G. R.: data collection and manuscript review; J. W. P.: study design and manuscript critical review; D. F.: study design, data analysis, manuscript writing and critical review; T. C.: study design, data analysis, manuscript writing and critical review; O. O.: study design, data collection and analysis, manuscript writing and critical review.

## Supplementary Data

Supplementary materials are available at *Open Forum Infectious Diseases* online. Consisting of data provided by the authors to benefit the reader, the posted materials are not copyedited and are the sole responsibility of the authors, so questions or comments should be addressed to the corresponding author.
