# Supplementary material for: Molecular Epidemiology of Isoniazid-resistant M tuberculosis in Port-au-Prince, Haiti
Source: Open Forum Infect Dis. 2024 Jul 18;11(8):ofae421. doi: 10.1093/ofid/ofae421 (PMC11306977; doi:10.1093/ofid/ofae421)

Supplementary Table 1. Univariate model to determine potential predictors of isoniazid-resistant TB among pulmonary TB patients in Port-au-Prince, Haiti

	Odds ratio	95% CI	p value
Sex (n=845)			
Female	Ref	Ref	-
Male	1.09	0.66, 1.84	0.734
Age, continuous variable (n=845)	1.01	0.99, 1.03	0.410
Age (n=845)			
Age ≥ 20	Ref	Ref	-
Age < 20	1.95	0.96, 3.69	0.049
Education (n=808)			
Primary school or less	Ref	Ref	-
Secondary school or higher	1.06	0.62, 1.78	0.839
Marital status (n=845)			
Divorced or separated	Ref	Ref	-
Single	1.01	0.34, 4.30	0.992
Married or living together	1.41	0.46, 6.16	0.588
Widower	3.00	0.51, 17.9	0.208
Missing data	1.27	0.22, 7.27	0.776
HIV status (n=845)			
Negative	Ref	Ref	-
Positive	1.39	0.67, 2.64	0.349
Prior INH use if HIV positive (n=111)			
No	Ref	Ref	
Yes	2.75	0.55, 11.1	0.174
Type of TB (n=845)			
First case	Ref	Ref	
Subsequent case	1.41	0.66, 2.76	0.341
Xpert level (n=845)			
Very low	Ref	Ref	-
Low	0.91	0.36, 2.49	0.844
Medium	0.60	0.26, 1.55	0.250
High	0.56	0.23, 1.51	0.220

Supplementary Table 2. ***M. tuberculosis*** **sub-lineages according to Coll scheme, stratified by isonia resistance as determined by Genotype MTBDR*plus***

Lineage	INH susceptible* n (%)	INH resistant* n (%)	P-value
4	3	1	0.623
4.1.1.3	9	1	0.023
4.1.2	1	1	1.000
4.1.2.1	28	23	0.636
4.3.2	2	1	1.000
4.3.4.1	6	18	0.011
4.4.1.1	3	4	0.983
4.8	10	7	0.627
4.9	1	1	1.000

*DNA from n=65 INH-susceptible and n=63 INH-resistant *M. tuberculosis* isolates underwent whole genome sequencing

Supplementary Figure 1. Phylogenetic tree of 128 *M. tuberculosis* strains from Port-au-Prince, Haiti with sub-lineages defined by different schemes and stratified by isoniazid resistance

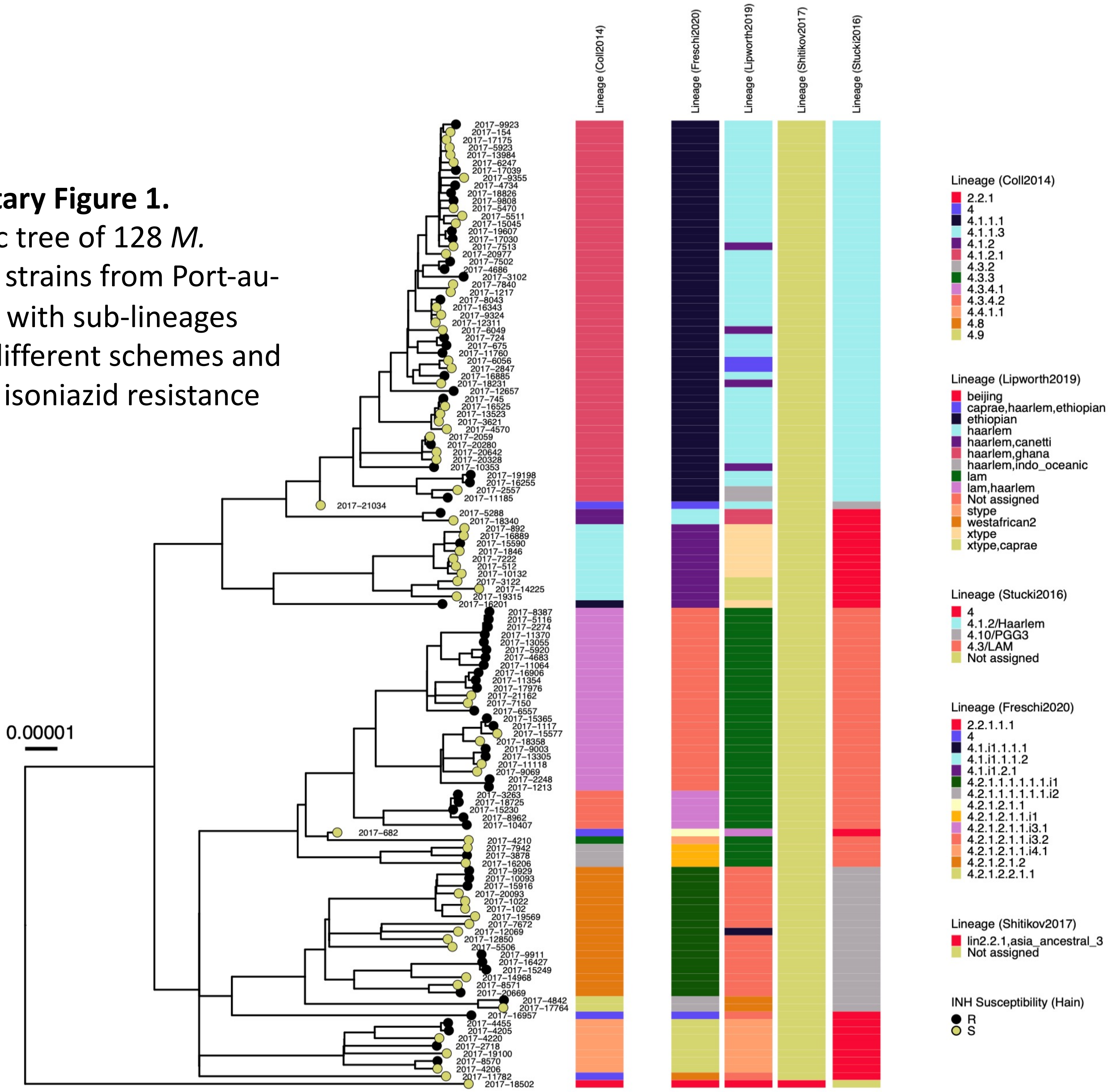

Supplementary Figure 2. Sub-lineages of 128 strains of *M. tuberculosis*, stratified by isoniazid resistance as determined by MTBDR*plus*

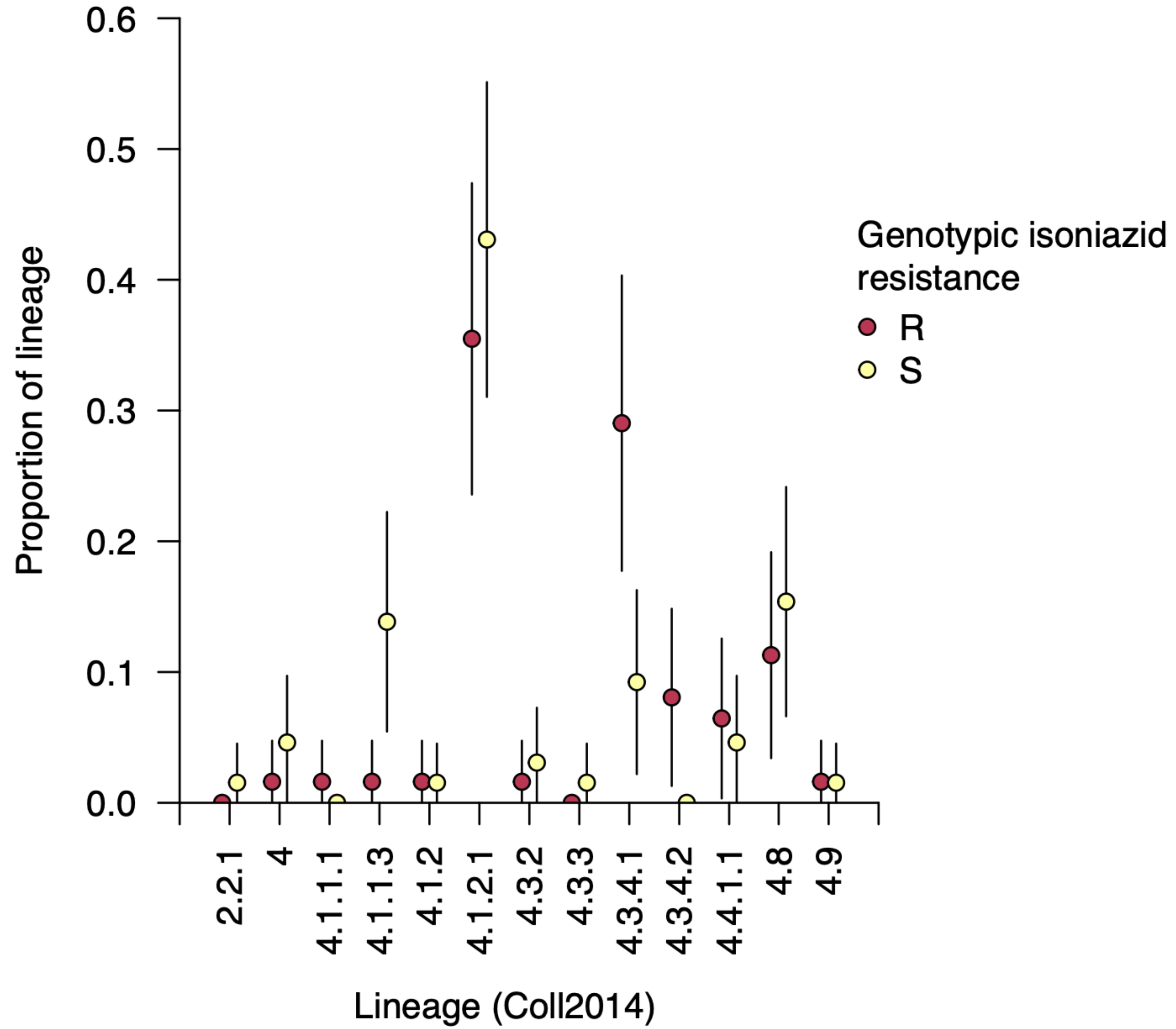

Supplement: ofae421_Supplementary_Data [file ofae421_supplementary_data.zip › Supplementary materials.pdf]
