# Supplementary figures and images for: Molecular Epidemiology of Isoniazid-resistant M tuberculosis in Port-au-Prince, Haiti
Source: Open Forum Infect Dis. 2024 Jul 18;11(8):ofae421. doi: 10.1093/ofid/ofae421 (PMC11306977; doi:10.1093/ofid/ofae421)

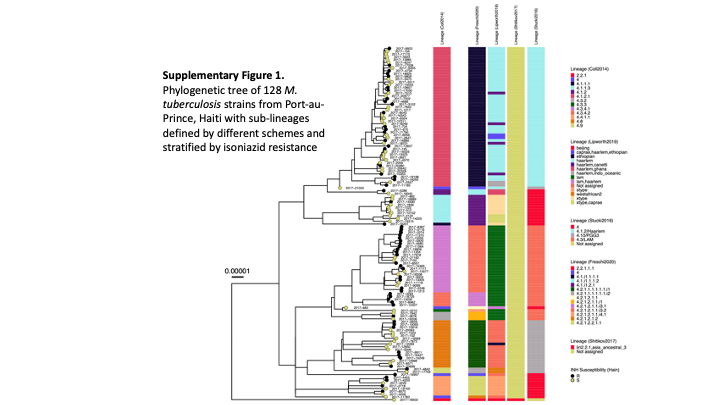

Supplement: ofae421_Supplementary_Data [file ofae421_supplementary_data.zip › Supplementary Figure 1_revised.tiff]
